# Interrelationships Among Men's Threat Potential, Facial Dominance, and Vocal Dominance

**DOI:** 10.1177/1474704917697332

**Published:** 2017-03-09

**Authors:** Chengyang Han, Michal Kandrik, Amanda C. Hahn, Claire I. Fisher, David R. Feinberg, Iris J. Holzleitner, Lisa M. DeBruine, Benedict C. Jones

**Affiliations:** 1 Institute of Neuroscience and Psychology, University of Glasgow, Glasgow, Scotland, UK; 2 Department of Psychology, Humboldt State University, Arcata, CA, USA; 3 Department of Psychology, Neuroscience, and Behaviour, McMaster University, Hamilton, Ontario, Canada

**Keywords:** dominance, voice, face, aggression, conflict, attractiveness

## Abstract

The benefits of minimizing the costs of engaging in violent conflict are thought to have shaped adaptations for the rapid assessment of others’ capacity to inflict physical harm. Although studies have suggested that men’s faces and voices both contain information about their threat potential, one recent study suggested that men’s faces are a more valid cue of their threat potential than their voices are. Consequently, the current study investigated the interrelationships among a composite measure of men’s actual threat potential (derived from the measures of their upper-body strength, height, and weight) and composite measures of these men’s perceived facial and vocal threat potential (derived from dominance, strength, and weight ratings of their faces and voices, respectively). Although men’s perceived facial and vocal threat potential were positively correlated, men’s actual threat potential was related to their perceived facial, but not vocal, threat potential. These results present new evidence that men’s faces may be a more valid cue of these aspects of threat potential than their voices are.

Evidence suggests that aggressive conflict among ancestral males has been an important selection pressure (Keeley, 1996; Manson et al., 1991) that may have led to adaptations for assessing the threat potential of others prior to actual combat (Puts, 2010; Sell et al., 2009). Much of the research into cues of threat potential in humans has investigated the relationships between the measures of men’s threat potential (e.g., measures of their upper-body strength, height, or weight) and their facial or vocal characteristics (reviewed in Puts, 2010; Puts, Jones, & DeBruine 2012).

Several studies have reported positive correlations between the measures of men’s upper-body strength (e.g., their handgrip strength) and ratings of their faces for dominance or strength (Fink, Neave, & Seydel, 2007; Holzleitner & Perrett, 2016; Sell et al., 2009; Windhager, Schaefer, & Fink, 2011). Strength ratings of men’s voices are also positively correlated with the measures of their actual physical strength (Sell et al., 2010), and men’s voices have been shown to contain acoustic characteristics that are correlated with their strength, height, and/or weight (Hill et al., 2013; Hodges-Simeon, Gurven, Puts, & Gaulin, 2014; Pisanski et al., 2016; Puts, Apicella, & Cardenas, 2012). Other work has found that taller men’s faces are perceived to be more dominant (Burton & Rule, 2013; Re, DeBruine, Jones, & Perrett, 2013). People can also predict the winners of mixed martial arts fights from facial cues alone at levels greater than chance (Little, Třebický, Havlíček, Roberts, & Kleisner, 2015). Collectively, these results suggest that both faces and voices contain cues of men’s threat potential. However, research reporting that men’s fighting ability can be assessed from their faces, but not their voices, suggests that faces may be a more valid cue of some aspects of men’s threat potential than their voices are (Doll et al., 2014).

In light of Doll et al.’s (2014) recent findings for fighting ability, we investigated the relationships among men’s actual threat potential and ratings of both their perceived facial and vocal threat potential. Men’s actual threat potential was assessed via a composite measure derived from a principal component analysis (PCA) of their handgrip strength, height, and weight. Perceived facial and vocal threat potential were assessed via composite measures derived from PCAs of dominance ratings, strength ratings, and weight ratings of their faces and voices, respectively. Men’s handgrip strength, face photographs, and voice recordings were collected on five separate occasions to ensure we obtained representative measures of men’s strength, facial appearance, and vocal appearance. Given Doll et al.’s (2014) findings, we predicted that the composite measure of men’s threat potential would be more strongly correlated with the composite measure of their perceived facial threat potential than with the composite measure of their perceived vocal threat potential.

## Method

Forty-four men (mean age = 22.02 years, *SD* = 3.41 years) each completed five weekly test sessions as part of a larger study on possible relationships among hormone levels and voice perceptions (Kandrik et al., 2016). In each of the five test sessions, each participant first cleaned his face with hypoallergenic face wipes. A full-face digital photograph was taken a minimum of 10 min later. Photographs were taken in a small windowless room against a constant background, under standardized diffuse lighting conditions, and participants were instructed to pose with a neutral expression. Camera-to-head distance and camera settings were held constant. Participants wore a white smock covering their clothing when photographed. Photographs were taken using a Nikon D300S digital camera and a GretagMacbeth 24-square ColorChecker chart was included in each image for use in color calibration. Following other recent work on social judgments of faces (e.g., Jones et al., 2015), face images were color calibrated using a least-squares transform from an 11-expression polynomial expansion developed to standardize color information across the images (Hong, Luo, & Rhodes, 2001). Images were masked, so that hairstyle and clothing were not visible and standardized in size and orientation on pupil positions.

In each of the five test sessions, a digital voice recording of each man was taken in mono using an Audio-Technica AT-4041 cardioid condenser microphone at a sampling rate of 44.1 kHz at 16-bit amplitude quantization. Each man was instructed to say “Hi, I’m a student at the University of Glasgow” in their normal speaking voice. The word “hi” was then extracted from each recording for use in the rating part of the study. The sound pressure level of all voices was amplitude normalized to 70 dB using the root mean squared method.

In each of the five test sessions, each man’s handgrip strength was measured 4 times using a T. K. K. 5001 Grip A dynamometer, alternating between the dominant (*M* = 42.15 kgf, *SD* = 7.84 kgf) and nondominant (*M* = 40.02 kgf, *SD* = 6.83 kgf) hand. Two men were left-handed and 42 were right-handed. In addition, each man’s height (*M* = 178.5 cm, *SD* = 6.75 cm) and weight (*M* = 72.65 kg, *SD* = 9.43 kg) was measured in one of the test sessions. Height, weight, and handgrip strength have been previously used to assess men’s threat potential (e.g., Puts, Apicella, & Cardenas, 2012).

Next, the face photographs of the 44 men (220 face photographs in total) and the voice recordings of the 44 men (220 voice recordings in total) were rated for dominance, strength, and weight using 1 (*low*) to 7 (*high*) scales. Faces and voices were presented in separate blocks of trials, and dominance, strength, and weight were rated in separate blocks of trials, respectively. Trial order was fully randomized within each block. None of the traits were defined for the raters and height was not rated. Thirty-two men and 47 women (mean age = 23.28 years, *SD* = 4.34 years) rated the faces and voices. The number of traits that each rater rated varied across raters. Each individual rater was randomly allocated to rate between two and four blocks of trials (mean number of raters per block of trials = 31.83, *SD* = 3.13). One rater chose not to report their age. Interrater agreement was high for all combinations of trait and stimulus type (all Cronbach’s αs > .89). Consequently, we calculated the mean dominance (face: *M* = 3.60, *SD* = 0.74; voice: *M* = 3.86, *SD* = 0.67), strength (face: *M* = 3.93, *SD* = 0.81; voice: *M* = 3.86, *SD* = 0.72), and weight (face: *M* = 4.26, *SD* = 0.83; voice: *M* = 4.01, *SD* = 0.59) rating for each man’s face and voice. Separate analyses of men’s and women’s ratings showed the same pattern of significant results as analyses of these combined ratings. Intercorrelations among ratings for each combination of trait and stimulus type across test sessions are given in our supplemental materials.

## Results

First, we subjected the ratings of men’s faces to PCA with no rotation. This analysis produced a single component that explained approximately 75% of the variance in scores and was highly correlated with facial strength (*r* = .98), dominance (*r* = .91), and weight (*r* = .67) ratings. We labeled this component the *perceived facial threat potential component*.

Next, we subjected the ratings of men’s voices to PCA with no rotation. This analysis produced a single component that explained approximately 82% of the variance in scores and was highly correlated with strength (*r* = .98), dominance (*r* = .88), and weight (*r* = .85) ratings. We labeled this component the *perceived vocal threat potential component*.

We also subjected our four measures of men’s threat potential (handgrip strength for dominant hand, handgrip strength for nondominant hand, height, and weight) to PCA with no rotation. This analysis produced a single component that explained approximately 60% of the variance in scores and was highly correlated with handgrip strength for nondominant hand (*r* = .93), handgrip strength for dominant hand (*r* = .89), weight (*r* = .68), and height (*r* = .54). We labeled this component the *actual threat potential component*.

Scores on the perceived facial threat potential component were positively correlated with scores on both the perceived vocal threat potential component (*r* = .37, *N* = 44, *p* = .012) and the actual threat potential component (*r* = .32, *N* = 44, *p* = .033). Scores on the perceived vocal threat potential component and the actual threat potential component were not significantly correlated (*r* = −.02, *N* = 44, *p* = .92). Steiger’s (1980) test showed that the correlation between the actual threat potential component and the perceived facial threat potential component was significantly stronger than the correlation between the actual threat potential component and the perceived vocal threat potential component (*z* = 1.97, *p* = .049). A table of intercorrelations among all variables is shown in our Supplemental Materials.

## Discussion

PCA of men’s handgrip strength, weight, and height produced a single “actual threat potential” component. This result is consistent with previous work, suggesting that these measures are positively correlated indices of men’s threat potential (Puts, Apicella, & Cardenas, 2012). Moreover, PCAs of the face and voice ratings each revealed a single perceived threat potential component. This result is consistent with previous research, suggesting that the impressions of men’s strength, dominance, and body size are positively intercorrelated (e.g., Holzleitner & Perrett, 2016). Further analyses showed that men’s perceived facial threat potential was positively related to their scores on the actual threat potential component. This result is consistent with previous research, suggesting that men’s faces contain cues to their actual threat potential (Burton & Rule, 2013; Doll et al., 2014; Fink et al., 2007; Hill et al., 2013; Holzleitner & Perrett, 2016; Re et al., 2013; Sell et al., 2009; Windhager et al., 2011). By contrast with our results for facial dominance, we found no evidence that people could judge men’s threat potential from their voices. Our results then complement those of Doll et al. (2014), who found that men’s fighting ability could be better assessed from their faces than their voices. While Doll et al. (2014) observed this pattern of results when men’s threat potential was measured from acquaintances’ ratings of their fighting ability, here we see the same pattern of results for the analyses of anthropometric measures of men’s threat potential. While some other studies with larger sample sizes have reported significant correlations between perceptions of men’s voices and measures of their threat potential (e.g., Sell et al., 2010), both our results and those of Doll et al. (2014) suggest that men’s faces are more valid cues to their threat potential than their voices are. Because Doll et al. used full sentences as their voice stimuli, the pattern of results that we observed in the current study is unlikely to be a consequence of the short snippets of speech we used as stimuli.

One recent study found that the ratings of men’s facial and vocal dominance were negatively correlated (Rezlescu et al., 2015). By contrast with Rezlescu et al.’s (2015) results, the current study found that men’s scores on the perceived facial and vocal threat potential components were positively and significantly correlated. In other words, our study found that men whose faces looked particularly dominant possessed voices that sounded particularly dominant. The positive correlation between facial and vocal threat potential observed in the current study is consistent with other research reporting correlations between perceptions of faces and voices (reviewed in Smith, Dunn, Baguley, & Stacey, 2016) and suggests that men’s faces and voices contain some overlapping information about their perceived threat potential. Our results suggest that the overlapping information in the perceived dominance of men’s faces and voices is unlikely to include information about their upper-body strength, height, or weight. It is possible that this correlation is driven by cues of men’s aggressiveness or emotional state (e.g., anger), rather than threat potential, per se.

In our study, participants rated the faces and voices for dominance, strength, and weight. It is possible that weight ratings of faces and voices are shaped by cues of adiposity, rather than formidability, per se. However, the results of our PCAs show that there is substantial overlap between weight, strength, and dominance ratings of men’s faces (see also voices). Thus, whatever information participants do use when they rate faces or voices for weight does seem to be highly correlated with the information that they use when making more direct assessments of formidability (strength and dominance ratings). A further unresolved question is what specific facial characteristics are valid cues of men’s threat potential. To date, most research addressing this question has focused on facial measurements of putative sexually dimorphic aspects of facial morphology. Such measures may be error prone in 2-D images, however, due to difficulties controlling for head tilt, among other factors (e.g., Schneider, Hecht, & Carbon, 2012).

In summary, we found that a composite measure of men’s actual threat potential (derived from measures of their upper-body strength, height, and weight) was positively correlated with a composite measure of these men’s perceived facial, but not vocal, threat potential (derived from dominance, strength, and weight ratings of their faces and voices, respectively). Together with Doll et al.’s (2014) results for men’s fighting ability, these findings suggest that men’s faces may be a more valid cue to some aspects of their threat potential than their voices are.

## Supplemental Material

Supplemental material for Interrelationships Among Men's Threat Potential, Facial Dominance, and Vocal Dominance Supplemental material, for Interrelationships Among Men's Threat Potential, Facial Dominance, and Vocal Dominance in Evolutionary Psychology
