# Supplementary material for: Interrelationships Among Men's Threat Potential, Facial Dominance, and Vocal Dominance
Source: Evol Psychol. 2017 Mar 9;15(1):1474704917697332. doi: 10.1177/1474704917697332 (PMC11383189; doi:10.1177/1474704917697332)
Supplement: Supplemental material for Interrelationships Among Men's Threat Potential, Facial Dominance, and Vocal Dominance [file sj-pdf-1-evp-10.1177_1474704917697332.pdf]

Supplemental Materials for "Interrelationships among men's threat potential, facial dominance, and vocal dominance"

Supplemental Materials 1. Inter-correlations among test sessions for each trait (table shows *r* values).

**Trait**	**Mean *r* value**	**SD**
Vocal weight	0.80	0.60
Vocal strength	0.84	0.78
Vocal dominance	0.78	0.74
Facial weight	0.96	0.84
Facial strength	0.96	0.84
Facial dominance	0.90	0.78

Supplemental Materials 2. Inter-correlations among all individual variables. Table shows *r* values (2-tailed *p* values in parentheses)

	Facial dominance rating	Facial strength rating	Facial weight rating	Vocal dominance rating	Vocal strength rating	Vocal weight rating	Height	Weight	Dominant handgrip strength
Facial strength rating	.92 (.001)								
Facial weight rating	.32 (.037)	.54 (.001)							
Vocal dominance rating	.14 (.354)	.22 (.159)	.09 (.555)						
Vocal strength rating	.33 (.028)	.40 (.007)	.18 (.251)	.86 (.001)					
Vocal weight rating	.39 (.009)	.49 (.001)	.34 (.024)	.53 (.001)	.80 (.001)				
Height	-.01 (.935)	.02 (.878)	.01 (.939)	.08 (.602)	.04 (.782)	.07 (.666)			
Weight	.15 (.320)	.32 (.033)	.78 (.001)	.02 (.878)	.04 (.783)	.28 (.063)	.25 (.107)		
Dominant handgrip strength	.20 (.196)	.26 (.090)	.23 (.137)	-.04 (.822)	-.13 (.389)	-.10 (.521)	.25 (.100)	.43 (.004)	
Non-dominant handgrip strength	.20 (.206)	.26 (.086)	.20 (.186)	-.07 (.640)	-.11 (.466)	-.02 (.904)	.38 (.010)	.46 (.002)	.87 (.001)
